# Socio-economic inequalities in ANC attendance among mothers who gave birth in the past 12 months in Debre Brehan town and surrounding rural areas, North East Ethiopia: a community-based survey

**DOI:** 10.1186/s12978-019-0768-8

**Published:** 2019-07-08

**Authors:** Gebretsadik Shibre, Wubegzier Mekonnen

**Affiliations:** Department of Reproductive Health and Health Services Management, School of Public Health, Addis Ababa University, Addis Ababa, Ethiopia

**Keywords:** ANC attendance, Socio-economic inequality, Decomposition, Debre Brehan;Ethiopia

## Abstract

**Background:**

In Ethiopia, socio-economic inequalities in the utilization of antenatal care (ANC) have long been an obstacle to the country’s effort in achieving universal coverage of the service. The study aimed to investigate socio-economic inequalities in the use of ANC services among recently-delivered women in Debre Brehan and surrounding areas, North East Ethiopia.

**Methods:**

A community-based survey was carried out in Debre Brehan and surrounding areas in North East Ethiopia. Two-stage cluster sampling technique was followed to recruit study participants. Data was collected using interviewer-administered structured questionnaire from a sample of 412 mothers who gave birth in the 12 months prior to the study. The socio-economic inequalities were assessed by calculating a relative concentration index. Decomposition analysis was done to explain measured inequalities. Analysis was carried out in RStudio statistical environment using the ‘decomp’ package.

**Results:**

The first ANC attendance has slight pro-poor concentration, with a relative concentration index of-0.128, and 95%CI -0.175, − 0.082.Socio-Economic Status (SES) of a household, educational level and occupation of a woman and her husband were the most important contributors to the measured inequality in ANC attendance. We found no SES-based inequality in the attendance of four or more ANC visits between the poor and rich.

**Conclusions:**

Attendance of the first ANC visit appeared to be slightly concentrated among women in the lower end of SES. The utilization of at least four ANC visits was found to be similar among the poor and rich. Population-based interventions that target all socio-economic groups are recommended to accelerate universal coverage of these process indicators.

## Plain English summary

Wealth-based inequality in the utilization of maternal health care in general and ANC in particular remains the biggest obstacle for Ethiopia to hit the 2030 Sustainable Development Goals. This study aimed to investigate the SES-related inequalities in the utilization of the first ANC visit and four or more ANC visits and to attribute the measured inequalities to various explanatory variables in North East Ethiopia. Existing evidence suggests huge poor-rich inequality in the use of basic maternal health care favoring the rich. However, all of those studies used the various waves of the Ethiopia DHS dataset which is not applicable to areas below regional states. Yet, context-specific evidence is necessary to formulate appropriate strategies to end poor-rich disparity in the use of ANC. Socio-economic status-based inequality in the use of the first ANC service was observed and was attributed to SES of a household, educational level and occupation of a woman and her husband. The study recommends launching strategies which target all population in the area to accelerate universal coverage of the above-mentioned maternal services.

## Background

Maternal death in Ethiopia continues to be one of the major public health problems for the country. In 2016, a Maternal Mortality Ratio (MMR) of 412 deaths per 100,000 births was reported [1], which is one of the highest even by the standard of developing countries [2]. This estimate of MMR positions the country among countries in the world which suffer from greatest maternal mortality burden [2]. Ethiopia is aiming to slash the current MMR to 140 deaths per 100,000 births within the next 12 years under the ambitious Sustainable Development Goals (SDG) [3]. Despite what the country had achieved under the completed Millennium Development Goal (MDG) [2, 4], this current pledge remains a serious challenge unless the country undergoes renewed movement in the pursuit of universal coverage of routine maternal health services such as skilled ANC attendance in all areas and regions of the country.

Provision of skilled ANC service is a useful intervention for boosting maternal health outcomes [5].The ANC service is an important contact point that brings receipt of the service and health professionals together, which in turn allows early detection and management of pregnancy related complications [6, 7]. This corresponds to lowering of the probability of death owing to unmanaged pregnancy-related complications [8].The ANC session also offers another imperative opportunity for skilled health professionals to professionally persuade an ANC attendee to come to a health facility to receive skilled delivery service [9], which itself has been linked to reduced maternal mortality during postpartum period [8]. Furthermore, the ANC service overcomes the inherent blemish of the commonly used maternal mortality outcome indicators in the measurement of progress towards the reduction of maternal mortality [10]. This means that skilled ANC service needs to be utilized by all women, irrespective of their wealth,in order for ANC to serve both of the aforementioned benefits. In this regard, the 2015 *Strategies toward ending preventable maternal mortality* reportfrom the World Health Organization highlights the need to abolish inequities in access to important strategies such as ANC in order to eliminate the occurrence of the preventable portion of maternal deaths [11].

However, economic inequality in skilled ANC service remains the main challenge in Ethiopia [12], with the service being mainly utilized by the rich people. To keep track of improvement of a certain health outcome, proxy indicators such as coverage rate of that health outcome could give us important clues as to whether we are heading to the set-forth goals pertaining to the health outcome in question [13]. However, overall coverage rates do not tell us the performances of sub-populations such as people in the poorest category of a wealth index. For instance, the current overall coverage for ANC service in Ethiopia is 62% for first ANC visit and 32% for at least four visits [1], with the prevalence differing based on the regions. Nonetheless, disaggregated analysis by socio-economic status (SES) shows huge inequality in the use of ANC service between the poor and rich [12], with people in the lower end of the socio-economic spectrum suffering from low coverage. The socio-economic inequality in the ANC service has persisted though the overall coverage for both indicators has steadily risen since 2000 [1]. This means that improvement in the average level of a health indicator could not eliminate inequality in the health indicator unless universal coverage is achieved. In Ethiopia, the national coverage gaps in first ANC visit and at least four visits of ANC are 38 and 68%,respectively [1]. The potential for improvement in the national coverage of both first ANC service and at least four visits of ANC if the sub-populations in all categories of wealth index had the same percentage coverage as the wealthiest sub-population for these health care services is respectively estimated at 41.1 and 26.9% [12]. These figures for the Population Attributable Risk (PAR) of ANC services highlight the need to have approaches that mainly target people from the poorest SES spectrum to efficiently improve these process indicators without ignoring the need to plan for the whole population approach.

There are few studies that have investigated the socio-economic inequality in the uptake of ANC in Ethiopia. Mezmur et al. [14] investigated the inequalities in ANC and other maternal health services using a concentration index based on the three rounds of EDHS between 2000 and 2011. The study revealed clear pro-rich concentration of both skilled ANC services. Gebre et al. [15] and Goli et al. [16] have each shown that the utilization of three or fewer ANC visits has a disproportionate concentration, with the poor using most of the service. However, this makes it impossible to guess concentration index of at least four ANC visits based on the calculated concentration index value of three or fewer ANC visits, since the standard concentration index does not have mirror property [17]. The study by Alam et al. [18] has confirmed the existence of socio-economic-driven inequality in at least four ANC visits, with the service dominated by people from the upper end of SES. All of the above-mentioned studies are based on data from the nationally representative Ethiopia Demographic and Health Surveys (EDHS). These conclusions could not, therefore, reflect the status of inequalities in ANC in areas such as the current setting. This is because DHS data is disaggregated to sub-national level such as region only, not to sub-national levels below regions. Within-country urban-rural disparity has a tremendous influence on utilization status of ANC and other maternal health care services [12], which necessitates carrying out small-scale studies in addition to the nationally representative surveys. An ANC inequality study that is confined to local area, such as the current setting, is urgently required to generate evidence that could be used to inform contextualized equitable strategies to help eliminate inequity in ANC between the poor and advantaged sub-populations. The paucity of national level and complete absence of local area studies on socio-economic status inequality in the utilization of ANC services underlie the motivation to undertake this study. Based on the already available evidence on inequality in ANC, two hypotheses are formulated here that the current study aims to prove or refute: 1) the use of the first ANC visit is relatively more concentrated among poor women and 2) the use of at least four ANC visits is relatively more concentrated among rich women. The objective of this study is therefore to assess wealth inequalities in the utilization of the first and four or more antenatal care visits in Debre Brehan and surrounding areas, North East Ethiopia.

## Methods

The study was conducted in Debre Brehan town and surrounding rural areas in North East Ethiopia. Debre Brehan was one of the ancient capital cities of Ethiopia and the Kingdom of Shewa. The town is now the capital city of North Shoa zone, Amhara National Regional State. Located 130 km north east of Addis Ababa, the town has an elevation of 2,840 m above sea level. Fourteen kebeles are under the jurisdiction of the town administration. Currently, there are one governmental referral hospital, one private general hospital, three health centers, and 6 different categories of private clinics. These facilities offer ANC services to the residents of the Debre Brehan town and referred clients from distant areas.

A community-based quantitative survey was undertaken among mothers who gave birth during the year preceding the survey. Single population proportion formula was used to estimate the sample size of study participants required, by considering 95% level of confidence, 5%margin of error, and50%ANC coverage in Debre Brehan town. The assumed 50% prevalence is for each of the first and at least four ANC services. The assumption behind the use of 50% is to determine the maximum possible sample size sufficient to capture both of these variables and improve representativeness of the sample to the wider population from which this sample has been drawn. Adding a non-response rate of 9% to the already-estimated sample size of 384, the final sample size is determined to be 418 mothers who delivered in the year prior to the study. Two-stage sampling technique was followed to draw eligible participants. In the first stage, six Kebeles (lowest administrative areas in Ethiopia) were randomly selected out of a total of 14 in the area using the simple random sampling method. In the second stage, households were drawn from each selected Kebele until the calculated sample size was reached. Each woman who had a delivery in the past year was interviewed at her usual place of residence using an interviewer-administered structured questionnaire. The interview took 10 to 15 min. The study was carried out between February 10 and February 25, 2018 and it took approximately two weeks to complete the survey with two interviewers.

### Outcome and explanatory variables

First and at least four skilled ANC services were the outcome variables for this study. Skilled ANC service is one that is offered by medical doctors, nurses, midwives, or health officers. Therefore, ANC service is said to be skilled if and only if it is provided by any one of these health professionals. Information about these ANC services are secured based on the woman’s self-report. The variable for first ANC visit was coded as 1 if the woman received ANC once from any one of the aforementioned health professionals and as 0 if they did not receive any ANC service. Similarly, at least four ANC service was coded as 1 if the women received ANC four or more times and 0 if they received ANC less than four times from skilled health care providers mentioned above. Both of the outcome variables are dichotomous. The classification of these outcome variables this way ensures that the variables are created in accordance with the recommendation of WHO [19].

The following explanatory variables were used in this study: mother’s age at last birth (17–19, 20–30 and 31–44), marital status (single, married and divorced/widowed), maternal education (primary, secondary, vocational and higher), husband’s education (primary, secondary, vocational and higher), maternal occupation (housewife, government employee, private employee, merchant, daily worker/student), husband’s occupation(government employee, private employee, merchant, daily worker/driver), time to reach nearest health centers (< 15 min, 15–29 min, 30–59 min and 1–2 h), time to reach nearest hospital (< 15 min, 15–29 min, 30–59 min and 1–2 h),birth order (1 and 2 or more), SES of the household (see below), insurance coverage(yes/no), religion (Orthodox, Catholic, Protestant, Muslim, traditional, others) and ethnicity (Amhara, Guraghe, Oromo, or Tigrie). For this study, health facilities refer to Health Centers (HC) and hospitals. The country’s health care system is structured in three tiered levels. The primary level includes five health posts (the lowest contact point that connects health care providers, i.e., Health Extension Workers, with clients), a health center and a primary hospital. The secondary level comprises a general hospital that offers health care services which the primary level could not normally provide. The tertiary level is where specialized services are provided. In relation to the ANC service, only the health centers and hospitals (both the general and tertiary) are eligible to provide skilled ANC (see the definition of skilled ANC service above).Health Extension Workers (HEWs) are not recognized as having enough proficiency to provide ANC service and are not included in our analysis. Concerning the ANC service, their responsibility is educating the community about the importance of giving birth at health facilities (HC and hospitals). In this study, the variables time to HC and time to hospital are separately entered in the analysis since time it takes to reach a hospital is usually greater than it takes to reach a HC. This is because hospitals are very few in number and are far away from the residence of most women, and only economically-advantaged women go there, which in turn affects the fair share of ANC between the poor and the rich.

### Measurement of socio-economic status

Wealth index was used to measure the household level SES of the studied samplesin accordance with a method recommended by Filmer and Pritchett [20]. We used principal component analysis to produce a wealth index variable. A varied set of household possessions and durable assets was used in the production of this composite variable. Household possessions and assets used in the calculation of wealth index were as follows: durable items (radio, television, refrigerator, table, chair, bed with mattress/sponge, electric grill (mitad), mobile telephone, fixed line phone, bicycle, motorcycle, vehicle, wall clock, DVD player, washing machine, computer/laptop, internet at home), kerosene lamp/pressure lamp electricity, water source for drinking, sanitation facilities, cooking fuel, materials used to make floor, roof and wall of the house, number of sleeping rooms and ability to hire maid servant. The generated wealth score was divided into five wealth quintiles: poorest, poorer, middle, richer and richest. For this study, “poor” and “rich” are understood by the concentration index and decomposition method as relative terms, with poor referring to poorest and poorer wealth quintiles and rich referring the richer and richest wealth quintiles. The women in the “middle” quintile can be counted as either poor or rich. Since women who fall under in any of the quintiles are not equally poor or equally rich, the “poor” or “rich” classification is merely a relative classification by considering the wealth index as a continuous variable though it is presented here as a categorical variable.

### Data collection procedures and assurance management

An interviewer-administered structured questionnaire was used to collect the data. The questionnaire was first developed in English and then translated into local Amharic language. Two Master’s degree public health professionals collected the data. Data collectors were trained on the techniques of data collection and/or face-to-face interview skills. The training also covered the importance of describing the purpose of the study to study participants. Coverage and content data analysis were done.

### Data analysis

The data were entered into EpiData version 3.1. Data was managed using STATA software v13. The prepared version of the data was transferred to RStudio statistical environment software for analysis. Owing to the binary nature of both of our outcome variables, the Generalized Linear Model (GLM)was fitted to measure inequalities and to subsequently decompose the measured inequalities to the underlying explanatory variables. RStudio statistical environment, together with the ‘decomp’ package, was used to calculate concentration indices and carry out the decomposition analysis.

### Inequality and decomposition analysis

The wealth-based inequalities in ANC attendance were quantified by calculatingits concentration index(C) [21]. This measure of inequality is already explained elsewhere [22–24]. In brief, the index is determined from the concentration curve. Plotted in the concentration curve are cumulative percentage of the variables of interest (in this study, first ANC and four or more ANC visits) on the y axis and the cumulative proportion of the sample population, ranked from the lowest to the richest wealth quintiles, on the x axis. Then, twice the area between the concentration curve and the diagonal line, that is, the line indicating perfect equality, defines the concentration index of the variables in question. The concentration index normally has values between − 1 and 1 inclusive. Whether the variables of interest are prevalent at one or another of the wealth quintiles based on the concentration index (C) and curve is decided in two ways: firstly, if the concentration curve lies above or below the line of no disparity, the variables dominated, respectively, the poor or the rich segments of the population. The second approach is to rely on the sign of the computed index; if C is positive, it means that the variable is concentrated among the wealthier group of people whilst negative C is indicative of pro-poor dominance of the health variable to be analyzed. The larger the concentration index in absolute value, the greater the socio-economic inequality is. If C is + 1, the health outcome variable of interest is totally concentrated among the rich sub-population. On the other hand, when C is − 1, the variable is entirely found among the poor. When C is zero, the concentration curve coincides with the 45-degree diagonal line, indicating perfect equality. Concentration index can be calculated through this formula [25]:

1 $$ \mathrm{C}=\frac{2\operatorname{cov}}{\upmu}\left(\mathrm{h},\mathrm{r}\right) $$

Where*μ* refers to the mean of our outcome variables (first and four or more ANC visits), *r* represents the socio-economic rank of the household, which in this study was proxied by wealth index, and *h* stands for the values of first ANC visit and four or more ANC visits for each participant.

Following computation of the concentration indices for each of the first ANC attendance and four or more ANC visits, the observed inequalities in these variables were decomposed into their sets of determinants through a decomposition analysis. The overall concentration index for the predicted outcome variables is computed as follows [25]:

2 $$ \mathrm{C}=\frac{\sum_{\mathrm{K}}\left({\upbeta}_{\mathrm{k}}{\mathrm{X}}_{\mathrm{k}}\right){\mathrm{C}}_{\mathrm{k}}}{\upmu}+\mathrm{GC}\upvarepsilon /\upmu $$

The formula explains that the predicted health outcome (first ANC visit and four or more ANC visits) is a result of summation of contributions made by all explanatory variables under investigation (k variables) [22]. Determinants contribute to the overall C through a combination of their concentration index (ck), regression coefficient (βk), and mean (xk), and the mean of the health outcome (μ) which was predicted by these determinants [26]. The GC*ε is the concentration index for the error term.* The (β_k_X_k_)/_μ_ C_k_ part in the formula represents the explained portion of the concentration index of the outcome variable, and the GC*ε* /μ refers to the residuals. The cut-off for declaring statistical significance was *p*-value<= 0.05.

## Results

The survey resulted in a response rate of 98.5%. Table 1 summarizes selected characteristics of the studied women. The number of women who used at least one ANC service for their last delivery was found to be around 386(93.7%). About 80% had received at least four ANC visits. Moreover, around the same percentage of studied women had initiated their ANC visit inside the recommended first four months of being pregnant. The remaining 20% had received ANC services after four months of gestation by the time they should have received two or more visits. All studied women were not covered by health insurance services. The dominant religion of the participants was Orthodox Christianity. Almost all (98%,401) of the respondents wereAmhara. Fifty-one (12.5%) of the women lived within a 15 min walk from the nearest hospital. 176(43%) of the interviewed women, the largest proportion, would need to walk between 30 and 59 min before reaching the closest hospital, with well above a quarter (115) required to walk for 15 to 29 min before reaching the nearest hospital. Only 16% (65) of them needed to travel for more than an hour to reach the nearest hospital. 27% (109) of the studied women reported that they travel for less than 15 min before reaching the nearest Health Center (HC), which is more than two-fold greater than the percentage of women who reported traveling for the same amount of time to reach the nearest hospital. Likewise, 46.7% (188) of women needed to travel between 15 and 29 min before reaching nearest HC, which is a higher proportion than that of women who spend the same time to reach the closest hospital. 13% (52) of the surveyed women needed to walk 30–59 min before reaching the nearest HC. The same percentage of women needed to travel 1 to 2 h before reaching the nearest HC. For nearly two-thirds of the study participants, a HC is available within less than half an hour of travel on foot, making the HC amore reachable health care delivery point than hospitals. This is not surprising as the study involves women who dwell in the urban setting. The proportion of households in each categories of the household level wealth is roughly 20%.

**Table 1:** Characteristics of the sampled women, Debre Brehan and surrounding rural areas,Ethiopia, 2018

Characteristics	Frequency	Percentage
Age of mother(*N* = 412)		
17–19	22	5
20–30	284	68.9
31–44	106	25.7
Birth order(*N* = 411)		
1	207	50.4
2 or more	204	49.6%
Education of mother(*N* = 389)		
Primary	124	31.88
Secondary	129	33.16
Vocational	78	20.05
Higher	58	14.91
Education of husband(*N* = 368)		
Primary	80	21.74
Secondary	114	30.98
Vocational	53	14.4
Higher	121	32.88
Marital status(N = 412)		
Single	17	4
Married	381	92.5
Divorced/widowed	14	3.4
Occupation of mother(N = 411)		
Housewife	229	55.7
Government employee	94	22.82
Private employee	16	3.88
Merchant	25	6.07
Daily worker/student	48	11.6
Occupation of husband(*N* = 398)		
Government employee	152	38.2
Private employee	99	24.87
Merchant	55	13.8
Daily worker/driver	92	23
Wealth index (*N* = 378)		
Poorest	77	20.4
Poorer	77	20.4
Middle	73	19.3
Rich	76	20
Richest	75	19.8

### Concentration index of first ANC visit and its decomposition analysis

**Fig. 1:**
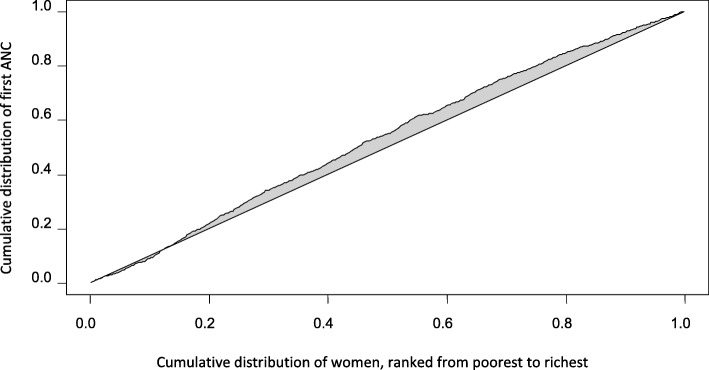
Concentration curve of the first ANC attendance, Debre Brehan, North East Ethiopia, 2018. The horizontal line that dissects the rectangle into two equal parts is the line of equality, i.e., the situation when all studied women use first ANC visit equally without any distinction based on their SES. The line that lies above this 45-degree line is the concentration curve, indicating more prevalence of the service among women from less well-off backgrounds. In the x axis of the graph, cumulative percentage of the study participant is plotted from the poorest to the richest wealth quintile. The y axis represents cumulative share of the first ANC attendance. Twice the area between the concentration curve and the equality line is concentration index

The relative concentration index (C) for first ANC attendance was found to be − 0.066, with variance of 0.0002. The 95% Confidence Interval (CI) was estimated and did not contain zero between its upper and lower limits: − 0.094, − 0.039.The negative concentration index of ANC attendance indicates that this maternal health care is concentrated among the worse off subgroups. We obtained the same conclusion of pro-poor dominance of ANC even after the potential effect of selected explanatory variables was adjusted in the logistic regression model.That is, the overall predicted C of ANC was − 0.128,with 95% confidence interval of − 0.175, − 0.082. The concentration curve for ANC (Fig. 1) supports this slight concentration of ANC services by women in the relatively less wealthy categories of wealth index. The curve lies above the diagonal line indicating that the poor were more likely to have used at least one ANC service than their counterparts in the upper end of socio-economic status.

Table 2 presents the individual contribution (result of decomposition analysis) of each explanatory risk factor to the observed wealth-based inequality in the uptake of ANC service. The table also contains information related to the regression coefficient and the concentration index of each risk factor of ANC, along with the statistics used to measure the associated significance (*p*-value and 95% CI).Women from households of higher Socio-Economic Status are more likely to use the service. Similarly, women who live close to health facilities are likely to go to these facilities to attend the ANC session. On the other hand, women who are salaried (both private and public) are less likely to utilize the skilled ANC service.

**Table 2:** Selected characteristics and their contribution to the inequality in first ANC attendance, DebreBrehan, Ethiopia, 2018

Variables	Coefficient	P-value	Contribution	Concentration Index (C)	95%CI for C
Wealth index			**46%**		
Poorest(ref)					
Poorer	22.6	0.99	85	−0.54	− 0.62, − 0.46**
Middle	3.03	0.13	2.5	−0.11	−0.2, − 0.016**
Rich	3.044	0.21	−7.5	0.33	0.24,0.42**
Richest	5.7	0.029*	− 34	0.78	0.73,0.82**
Age of mother			**−1.99%**		
17–19(ref)					
20–25	−0.098	0.95	− 0.05	− 0.056	− 0.15, 0.04
26–30	2.8	0.12	−0.74	0.0186	−0.055, 0.09
31–44	2.73	0.16	−1.2	0.056	−0.06,0.175
Birth order			**1.14%**		
1(ref)					
2	0.39	0.84	−0.16	0.04	− 0.06,0.14
3	−3.5	0.056	2.26	0.13	−0.018, 0.28
4 or more	−5.1	0.081	−0.96	−0.12	− 0.44,0.2
Marital status			**6.78%**		
Single(ref)					
Married	−22.6	0.998	10.9	0.015	−0.0003,0.03
Divorced/widowed	−41	0.999	−4.12	−0.47	−0.79,-0.15**
Education of mother			**−13.61%**		
Primary(ref)					
Secondary	1.5	0.389	−1.56	0.09	0.008,0.17**
Vocational	5.3	0.071	−5.9	0.16	0.033,0.29**
Higher	6.6	0.069	−6.15	0.175	0.002,0.35**
Education of husband			**29.56%**		
Primary(ref)					
Secondary	28.9	0.991	18.7	−0.059	−0.15,0.03
Vocational	−0.14	0.919	0.056	0.073	−0.084,0.23
Higher	−3.5	0.162	10.8	0.28	0.2,0.367**
Occupation of mother			**9.6%**		
Housewife(ref)					
Government employee	−6.5	0.0398*	4.77	0.088	−0.03,0.21
Private employee	−14	0.0391*	5.57	0.26	−0.02,0.54
Merchant	−4.03	0.0859	3.23	0.33	0.14,0.52**
Daily worker	0.024	0.9881	0.033	−0.58	−0.71,-0.44**
Student/other	19.6	0.999	−4.02	0.48	0.19,0.77**
Occupation of husband			**27%**		
Government employee(ref)					
Private employee	−3.5	0.057	2.4	0.07	−0.03,0.17
Merchant*	−8.4	0.031	9.5	0.022	0.08,0.35**
Daily worker	14.5	0.998	21	−0.378	−0.5,-0.256**
Driver/student	−5.35	0.0769	−5.9	−0.45	−0.66,-0.24**
Time to reach hospital			−**21.096**		
Less than 15 min(ref)					
15–29 min	−11.2	0.0557	9.9	0.092	−0.0074,0.19
30–59 min	−15.6	0.0518	−31	−0.13	− 0.198,-0.06**
1–2 h	−12.4	0.0643	0.004	0.000073	−0.16,0.16
Time to reach HC			**16.18%**		
Less than 15 min(ref)					
15–29 min	7.1	0.0479*	13	−0.11	− 0.18,-0.05**
30–59 min	6.5	0.0685	2.8	−0.104	− 0.27,0.06
1–2 h	3.4	0.3468	0.38	−0.03	− 0.2,0.14

* Indicates variables which have significant regression coefficient at < 0.05 p-value; ** indicates variables whose concentration index is different from zero by 95%CI; CI=Confidence Interval; ref.: referent group; HC: Health Center

The table portrays the decomposition analysis outputs of the Generalized Linear Model for the first ANC attendance. Variables (explanatory or independent variables) are presented in the first column of the table. The second column describes the regression coefficient of each variable with the first ANC attendance. Statistically significantly associated variables at P-value < 0.05 are marked with asterisk symbol (third column). The fourth column shows how much each explanatory variable contributes (in percent) to the inequality. In the fifth column, information related to concentration index of each independent variable is presented. This index measures how unequally are the independent variables distributed across the socio-economic sub-populations. Whether this unequal distribution is not by chance is measured by calculating the 95% uncertainty interval around the point estimates (sixth column)

To facilitate interpretation of the contribution of each of the explanatory variables to the inequality in ANC, it is important to first see these variables’ distribution along the SES spectrum based on their concentration index. A variable contributes to an inequality if it is unequally distributed (which is reflected by its concentration index) between the poor and rich people. For instance, mother’s secondary or above level of schooling, husband’s higher level of education, mother’s occupation (being merchant or student) and occupation of husband (being merchant) are all concentrated among people of higher SES (they have positive concentration index). In contrast, women whose marital status fall in the category of divorced/widowed, daily worker women, daily worker husbands, and driver husbands or students are all negatively associated with SES, i.e., they are relatively positioned at the lower end of the socio-economic rank. This is reflected by their concentration index being negative. Wealth index itself has different concentration indices for its different categories. The ‘poorer’ and ‘middle’ categories have negative concentration index indicating that women in this category are really less well off economically, whereas the ‘richest’ sub-group has positive concentration index meaning that people in this category are indeed relatively better off.

The largest contributions to the observed wealth-based inequality in ANC attendance were attributable to the household level wealth (46%), educational status of the husband (30%), and occupation of the husband (27%), health facility-related factors (21%), educational level of the women (14%) and occupation of the women (10%).

### Concentration index of four or more ANC visits and its decomposition analysis

The adjusted associations (in terms of regression coefficients) between explanatory variables and four or more ANC visits are presented in Table 3. Educational status (secondary level) and occupation type (driver or student and employed in private organization) of the husband are both negatively associated withfour and more ANC visits.

**Table 3:** Selected characteristics and their contribution to the inequality in at least four ANC visits, DebreBrehan, Ethiopia, 2018

Variables	Coefficient	P-value	Contribution	Concentration Index (C)	95%CI for C
Wealth index					
Poorest(ref)					
Poorer#	−0.17	0.8	−18.6	− 0.54	− 0.62,-0.46
Middle#	− 0.69	0.32	−16.7	−0.11	− 0.21,-0.02
Rich#	−0.03	0.96	2.4	0.33	0.24,0.42
Richest#	0.22	0.78	−37.2	0.775	0.73,0.82
Age of mother					
17–19(ref)					
20–25	1.07	0.13	17.4	−0.056	−0.15,0.04
26–30	1.04	0.14	− 8.1	0.019	−0.05,0.09
31–44	0.76	0.37	−10	0.056	−0.06,0.18
Birth order					
1(ref)					
2	0.03	0.95	−0.4	0.04	−0.06,0.14
3	0.07	0.91	−1.3	0.13	−0.02,0.3
4 or more	−0.06	0.944	−0.34	−0.12	− 0.44,0.2
Marital status					
Single(ref)					
Married	−15.7	0.99	220	0.015	−0.0003,0.03
Divorced/widowed#	−18.3	0.98	−53.3	−0.47	−0.79,-0.15
Educationof mother					
Primary(ref)					
Secondary #	−0.07	0.88	2.1	0.09	0.008,0.17
Vocational#	0.088	0.9	−2.9	0.16	0.033,0.3
Higher #	0.60	0.4	−16.4	0.18	0.0022,0.35
Education of husband					
Primary(ref)					
Secondary	−1.8	0.0059*	−34.3	−0.06	−0.15,0.03
Vocational	−1.007	0.22	11.3	0.073	−0.08,0.23
Higher#	−1.3	0.099	115	0.28	0.2,0.367
Occupation of mother					
Housewife(ref)					
Government employee	−0.778	0.19	16.5	0.088	−0.03,0.21
Private employee	−0.08	0.93	0.93	0.26	−0.016,0.54
Merchant#	1.16	0.18	−27	0.33	0.14,0.52
Daily worker#	−0.04	0.95	−1.96	−0.58	− 0.71,-0.44
Student/other#	15.7	0.99	−94	0.48	0.19,0.77
Occupation of husband					
Government employee(ref)					
Private employee	−1.2	0.015*	23.6	0.07	−0.03,0.17
Merchant#	−1.1	0.07	36	0.22	0.08,0.35
Daily worker#	0.45	0.63	18.8	−0.378	−0.5, − 0.256
Driver/student#	−1.66	0.04*	53.5	−0.45	−0.66,-0.24
Time to hospital					
Less than 15 min(ref)					
15–29 min	0.6	0.36	−15.6	0.09	−0.007,0.19
30–59 min#	0.63	0.48	36	−0.13	− 0.197,-0.06
1–2 h	−0.79	0.55	0.0076	0.000073	−0.16,0.16
Time to health center					
Less than 15 min(ref)					
15–29 min#	−0.09	0.9	−4.6	−0.11	− 0.17,-0.04
30–59 min	−0.72	0.40	−9.1	−0.1	− 0.27,0.06
1–2 h	1.3	0.35	4.2	−0.028	− 0.2,0.14

* Variables with non-zero coefficients at < 0.05 p-value

^#^Variables with non-zero concentration index by 95%CI; CI = Confidence Interval; ref.: referent group

The table portrays the decomposition analysis outputs of the Generalized Linear Model for the attendance of at least four ANC visits.Variables (explanatory or independent variables) are presented in the first column of the table. The second column describes the regression coefficient of each variable with the attendance of at least four ANC visits. Statistically significantly associated variables at P-value < 0.05 are marked with asterisk symbol (third column). The fourth column shows how much each explanatory variable contributes (in percent) to the inequality. In the fifth column, information related to concentration index of each independent variable is presented. This index measures how unequally are the independent variables distributed across the socio-economic sub-populations. Whether this unequal distribution is not by chance is measured by calculating the 95% uncertainty interval around the point estimates (sixth column)

**Fig. 2:**
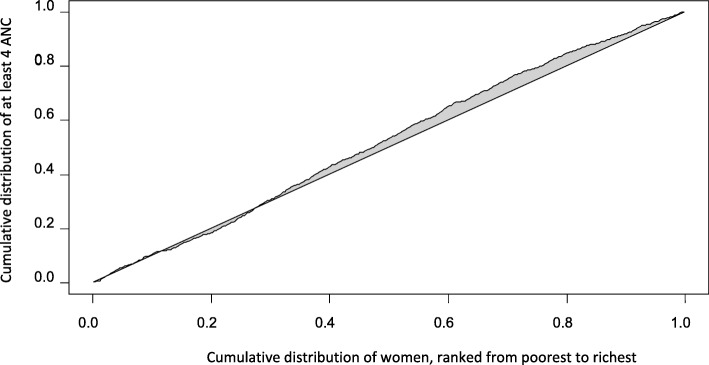
Concentration curve of four or more ANC visits, Debre Brehan, Ethiopia, 2018. The horizontal line that dissects the rectangle into two equal parts is the line of equality, i.e., the situation when all studied women use at least four ANC visits equally without any distinction based on their SES. The line that lies above this 45-degree line is the concentration curve, indicating more prevalence of the service (based on unadjusted regression) among women from less well-off backgrounds. In the x axis of the graph, cumulative percentage of the study participant is plotted, beginning from the poorest to the richest wealth quintile. The y axis represents cumulative share of the attendance of at least four ANC visits. Twice the area between the concentration curve and the equality line is concentration index

The crude relative concentration index (C)for four or more ANC visits is − 0.04, with a 95%CI of − 0.07, − 0.015and variance of 0.000195.The C being negative proves that this basic maternity care is more likely to be utilized by women from a household with higher SES. The same conclusion is drawn by referring to a concentration curve (Fig. 2) inequality measure, where the curve remains above the 45-degree line throughout, indicating that the service is more concentrated among less well-off people. However, the regression-based overall predictedC of this skilled service is − 0.023, with 95% confidence interval of − 0.08, 0.035.Clearly,the uncertainty interval for C crosses zero and therefore there is no sufficient reason to conclude that the attendance of four or more ANC visits has shown a pro-poor dominance in relation to the socio-economic rank. Still, we decomposed the concentration index of this maternity care based on the hypothesis that there exists inequality in the use of four or more ANC services based on evidence from the crude concentration index and we presented the outputs in Table 3. Secondary or higher educational status of the mother, higher education level of the husband, certain types of occupation of the parents (e.g. merchant) are positively associated with economic status rank, i.e., they are more concentrated among the better off people. Other conditions such as being reliant on daily work as source of income and time it takes to reach health facility aremore concentrated among the lower end of the SES.In the SES itself, the ‘poorer’ and ‘middle’ categories have negative concentration index, and are therefore reallyrelatively poor, and the opposite is true for the ‘rich’ and ‘richest’ categories of wealth index.

Despite the unequal distribution of the aforementioned variables along the SES rank, these unequal distributions did not bring inequality in the attendance of four or more ANC visits.

## Discussion

The study assessed socio-economic inequality in the utilization of ANC services using the concentration index method. Concentration index has been the dominant summary measure in health economics literature. Wagstaff et al. [27] have discussed the important attributes of a health inequality index: reflecting the socio-economic dimension to health disparity, taking into account the entire sub-population and being sensitive to a change in the distribution of the entire population across the socio-economic groups. Concentration index has met all the three properties of a rigorous inequality measure and help address the inherent limitations of the traditional inequality measures such as ratio and difference.

The overall coverage rates for both ANC visit variables were reasonably high. The improved aggregate percentage level of ANC could be attributed to the recent revitalized move in extensively engaging Urban Health Extension Workers (HEW) [28] in the health care delivery system of the country. In the three-tiered health care delivery system of the country, HEW and the Health Extension Program (HEP) constitute the lowest point of contact between the clients and the health care providers [29].The lowest point of contact is the entry point where clients formally contact health care providers for the first time before going to the next higher level of health care. This allows the HEWs to work closely with the community. Owing to their high acceptance by the community, they are likely to persuade the community about the benefits of going to health facilities for ANC, which in turn improves its coverage [28].

Despite high coverage, the survey clearly unveiled the presence of socio-economic inequality in the uptake of first ANC attendance to the advantage of the poor, proving the hypothesis that first ANC attendance has pro-poor tendency. Both the concentration index (negative) and concentration curve (lies above the ‘no inequality’ line) inequality measures proved the service to be relatively more concentrated among women in the lower end of economic status. This implies that the sheer high coverage rate for a health outcome indicator does not warrant absence of inequality in the health indicator unless universal coverage is achieved. The concentration index being negative for the first ANC visit in this study is consistent with findings of other studies [15, 16] where fewer than three ANC attendances was more common among poorer women. However, this conclusion is inconsistent with Mezmur et al. [14], who reported increased use of first ANC visit by economically-advantaged people. ANC service in Ethiopia is among the list of a few interventions waived from any professional charges related to the service, making it easy for people from any socio-economic background to utilize the skilled care. Attending a first ANC visit might not be difficult even for poor women as long as they do not incur any additional costs. It is likely that health care facilities become clustered in urban areas, which leads to improved access to a wide range of maternal health care including ANC for the people who dwell in these areas [18], even when they are from the lower SES. The current setting being predominantly urban and semi-urban partly explains the highest utilization of ANC by the women from poorer end of the SES, which is consistent with findings from the EDHS-based studies.

The underlying inequality in economic status, education status and occupation of the mother and her husband have explained the largest part of the observed inequality in first ANC attendance from among all variables used in the logistic regression model. In support of the current finding, available evidence [14, 15] has shown that SES disparity has been the leading contributing factor of disparity in the use of ANC service, irrespective of whether the inequality is pro-poor or pro-rich. In this study, educational status and occupation of a husband of the woman had positive contribution, with both of these variables being more prevalent among women from well-to-do families (have positive concentration index). This implies that these variables operate by preventing the rich people from accessing the services, which in turn resulted in raised inequality to the favor of the poor. Previous studies have confirmed the combined 18% contribution of the women’s education and occupation to the disparity in ANC [15]. Another study has similarly shown that education of a woman was found to have noticeably caused poor-rich inequality in ANC [14].Both of these studies used data from the series of EDHS conducted at different points in time between 2000 and 2016. They did not include education and occupation characteristics of the husband of the woman in their analysis, failing to appreciate the individual influence of these important social variables on the utilization disparity of ANC. Yet, husband-related variables are important factors affecting the utilization of maternal ANC service [30].

Another reason for the first ANC visit to be slightly dominated among the poor is that women who have to travel 15 to 29 min before getting ANC services were found to be poor (negative concentration index) but had positively contributed to the inequality, i.e., it helped them (the poor) to access the service more easily. Also, women who rely on daily work and trade helped to widen the inequality, with each having a different mechanism. Daily worker women were relatively poor (had negative concentration index) and were more likely to use the service, and merchant women were relatively rich (positive concentration index) and were less likely to utilize the ANC service. This differential influence on the inequality led to the raised socio-economic inequality in ANC, with the poor having overall higher use of ANC. This phenomenon clearly supports the fact that a variable contributes to the widening or narrowing of an inequality if the variable itself is unequally distributed along the SES [26]. Other important contributors to the disparity included time it takes to travel to a health facility, marital status and birth order. Existing literature similarly found that birth order contributed to the observed inequality in ANC service but marital status was found not to play a role in causing the inequality [15].

Some of the above-mentioned explanatory variables have positive contribution, meaning that they are likely to raise the poor-rich gap in the use of ANC. Others determinants have negative contribution, which means that they operate by narrowing the observed inequality. Whether the aforementioned independent factors favor the poor or the rich in the use of the service is a function of their concentration indices and contributions (negative or positive). For instance, educational level of a mother contributes negatively to the inequality, and this reduces the inequality by helping the rich to access ANC because mothers with secondary or post-secondary education are likely to be rich (positive concentration index). This means that since ANC is already more concentrated among the poor, the poor-rich gap in using ANC decreases as the more affluent mother tended to use more of the service. The striking finding in relation to marital status was that divorced and widowed women were poor (as reflected by negative concentration index) and contributed to the inequality negatively. This occurred because these women did not use skilled ANC service.

The study demonstrated that utilization of four or more ANC visits was uniformly distributed between the poor and rich, and refuted our hypothesis that four or more ANC is mainly utilized by women who are rich. Equal utilization of this life-saving maternal health service is crucially important to battle the unacceptably high maternal mortality burden in the country [1, 19] and subsequently helps to attain the 2030 SDG by “leaving no women behind” [3]. The 2016Lancet report on the Global Burden of Disease study has shown that 91% coverage rate for first ANC visit, 78% coverage for at least four ANC visits and 87% coverage of skilled birth attendance are required to successfully hit the 2030 SDG 3.1 [31]. The evidence highly underscores the unequivocal importance of universal coverage of the process indicators in the decline of maternal deaths. Attending a minimum of four ANC sessions is likely to increase the provider-ANC attendee interaction, there byincreasing the possibility of early detection and timely management of deadly pregnancy complications [19].In view of the newest ANC recommendations of the WHO [32], the estimate of ANC coverage for the current study setting is reflective of poor performance of the country given the SDG 3.1.

Since a high coverage rate for at least four ANC visits in a population does not guarantee that there is no inequality, it is always necessary to undertake inequality analysis in the use of a health outcome unless the health outcome of interest is utilized by the whole population, i.e., universally available to all who need it. We often use outcome indicators such as MMR and lifetime risk of maternal deaths (LFR) to measure maternal mortality burden in developing countries. However, these outcome indicators suffer certain inherent problems in properly indicating whether we are heading to the set-forth goal [10]. Process indicators like attendance of at least four ANC visits have useful implications in overcoming the intrinsic blemish of those outcome indicators in showing the real progress towards reducing maternal mortality.

The absence of wealth-based inequality in the use of at least four ANC visits in this study is contrary to that of the existing EDHS-based literature, which demonstrated SES-based inequality in this service favoring the better-off [18]. The reasons might be justified by the fact that efforts have been made(both nationally and locally) over the more than ten years since the conduct of that study, which could help eliminate unjust inequalities in the utilization of the ANC service. As well, the urban HEWs regularly trace the ANC defaulters in the community and advise the defaulters to continue the remaining ANC visits. This active community-based detection and referral of the defaulted mothers to facilities could have largely contributed to the equitable distribution of the utilization of at least four ANC services in the study area.

Imperative policy implications emerge from this study. Since the greatest portion of the measured inequality in the attendance of first ANC visit was accounted for by SES disparity, eliminating inequality around utilization of maternal health care services is better done by a determined partnership between the Ministry of Health and Ministry of Agriculture. The absence of inequality in the attendance of at least four ANC visits and presence of pro-poor dominance in the use of the first ANC visits uggest that it is worth considering the whole population approach that targets all population in the area to achieve universal coverage for both ANC services. The “leaving no one behind” slogan of the SDG 3indicates the need to reach all women, irrespective of their SES backgrounds, with basic maternity care including ANC.

Despite its meaningful contribution to policy making, the study suffers from a few limitations. The mainly urban setting makes it difficult to generalize findings to the wider rural population as the ANC utilization varies by the rural-urban disparity [18]. Wealth index was computed to serve as a proxy measure of household level SES, and the computation of wealth index was based on household characteristics and durable assets. Some household characteristics such as TV, radio and materials the household is made from might not necessarily accurately measure the socio-economic rank of the households, thereby inaccurately classifying them as poorest, poorer, middle, rich or richest.

Future studies are recommended to further understanding of the nature of inequity of ANC use in the studied community. Particularly important is identifying inequity in ANC quality, as the mere existence of the physical service does not guarantee provision of quality service equitably between the poor and the rich. Improvements of quality gaps in ANC between the poor and rich could help decrease maternal health problems attributable to the inequitable distribution of poor ANC service.

## Conclusions

The study sheds light on the slight pro-poor situation in the use of the first skilled ANC visit. Inequalities in economic status, education status and occupation of the mother and her husband have explained the largest part of the measured SES-based inequality in the utilization of first ANC attendance. We did not discover SES-based inequality in the use of four or more ANC services.

## Data Availability

All data analyzed during this study are included in this published article.

## Abbreviations

ANC: Antenatal care
C: Concentration index
EDHS: Ethiopia Demographic and Health Survey
HEP: Health Extension Program
HEW: Health Extension Worker
SDG: Sustainable Development Goal
SES: Socio-Economic Status
